# Effects of physical activity on anxiety phenomenon in vocational college students–chain mediation of self-control and mobile phone dependence

**DOI:** 10.3389/fpsyg.2026.1786180

**Published:** 2026-04-17

**Authors:** Jiahui Dong, Yan Shi, Bingzhi Wan, Teng Ding, Qi Zhang

**Affiliations:** 1 School of Physical Education, Shaanxi Normal University, Xi'an, China; 2 Faculty of Education, Shaanxi Normal University, Xi'an, China; 3 Department of Physical Education, Xidian University, Xi'an, China; 4 School of Physical Education, Ningxia University, Yinchuan, China; 5 Department of Physical Education, Yangling Vocational and Technical University, Xianyang, China

**Keywords:** vocational college students, physical activity (PA), anxiety (AD), self-control (S-C), mobile phone dependence (MPD)

## Abstract

**Background:**

Anxiety has emerged as a significant concern among students at vocational colleges in China and has received sustained attention from scholars in sports science. This study’s primary objective was to examine the relationships among physical activity, self-control, mobile phone dependence, and anxiety in vocational college students within a digital society. The secondary objective was to assess the specific roles of mobile phone dependence and self-control in relation to anxiety.

**Methods:**

A cross-sectional survey used the Physical Activity Rating Scale, Self-Control Scale, Mobile Phone Dependence Index, and Anxiety Scale. The sample comprised 5,991 vocational college students in Shaanxi Province (male: 3,443; female: 2,548; mean age = 19.23, SD = 0.91). Descriptive analyses summarized demographic variables. *Spearman* correlation, regression, and chain mediation analyses were applied to model relations among the variables.

**Results:**

Physical activity correlated positively with self-control, and both variables correlated negatively with mobile phone dependence and anxiety. Mobile phone dependence correlated positively with anxiety.

**Conclusion:**

Self-control and mobile phone dependence each independently mediated the relationship between physical activity and anxiety. In addition, they operated as sequential (chain) mediators linking physical activity to anxiety among vocational college students.

## 1 Introduction

As digital society advances, the deep integration of smartphones has increased convenience but also raised widespread concern about mental health. Non-substance addictive behaviors, particularly mobile phone dependence, have become a major public-health challenge and are often comorbid with anxiety and depression. This pattern is pronounced among vocational college students, who exhibit relatively high detection rates of mobile phone dependence and elevated anxiety levels (Xiaoyu et al., 2023; Yaoxiu et al., 2020), indicating heightened mental-health risk in this group. Mobile phone dependence disrupts academic and daily functioning and, more importantly, erodes psychosocial adaptation (Elhai et al., 2017; Jianguo, 2009). Thus, identifying effective interventions for mobile phone dependence and its associated emotional distress is of substantial practical significance.

### 1.1 The relationship between physical activity and anxiety

The relationship between physical activity and anxiety is central to sports psychology and public health. Extensive research has established regular physical activity as a protective factor that both alleviates and helps prevent anxiety, with mechanisms that can be understood at physiological, psychosocial, and cognitive-neuroscientific levels.

From physiological and neurochemical perspectives, physical activity directly modulates neuroendocrine systems involved in emotion. It promotes the release and balance of neurotransmitters such as dopamine, serotonin, and endorphins, which are central to mood regulation, generate pleasure, and alleviate tension, thus providing a physiological basis for anxiety reduction (Wei, 2025). At the psychosocial and behavioral level, physical activity acts through multiple mechanisms. First, as a form of positive behavioral activation, it disrupts the avoidance–withdrawal cycle often seen in anxiety and helps individuals re-engage with and regain control over their lives. Second, successfully meeting physical challenges can increase self-efficacy and foster psychological resources such as resilience and hope, which protect against anxiety (Meng-long et al., 2020). Third, group-based or social physical activity enhances interpersonal interaction and social support, strengthening social connectedness and buffering anxious feelings. From a cognitive neuroscience perspective, anxious individuals frequently show an attentional bias toward negative information. Physical activity has been shown to improve attentional control, especially the ability to disengage from negative stimuli, thereby reducing the cognitive bias that sustains anxiety; attentional bias partially mediates the anxiety-reducing effects of physical activity (Zhifeng et al., 2024).

In summary, physical activity is a multifaceted intervention that integrates physiological regulation, behavioral activation, cognitive restructuring, and social support. Among vocational college students, physical fitness levels are closely linked to mental health status (Manluo et al., 2025; Weimin and Zufeiya, 2024; Yin et al., 2022). Based on this, the present study proposes the following hypothesis:

*H1*: Physical activity can directly and negatively predict the anxiety levels of vocational college students.

### 1.2 The mediating role of self-control between physical activity and anxiety among vocational college students

The mediating role of self-control in the association between physical activity and anxiety provides a key pathway for understanding the internal mechanisms by which exercise benefits mental health. This relationship is supported by both theoretical and empirical evidence.

Theoretically, self-control is framed as a limited psychological resource that can be depleted yet strengthened through training (the strength model) (Baumeister and Tice, 2007), and it also functions as a central capacity for emotion regulation. High self-control enables individuals to inhibit impulses more effectively, reduce negative rumination, and adopt adaptive coping strategies, thereby directly buffering anxiety (de Ridder et al., 2012; Tangney et al., 2004). Physical activity provides a systematic route to enhance self-control. Physiologically, regular exercise increases release of neurotransmitters involved in reward and emotion regulation and bolsters prefrontal cortex function, offering a neural substrate for cognitive control (Mandolesi et al., 2018; Weinstein and Weinstein, 2014). Behaviorally, participation in physical activity requires adherence to rules, goal setting, and overcoming physical and mental discomfort; this repetition strengthens perseverance, delayed gratification, and psychological resilience, and thus directly improves self-control (Boat and Cooper, 2019; Friese et al., 2017).

This relationship can be described by a clear transmission chain: regular physical activity strengthens an individual’s self-control capacity. Improved self-control enables more effective cognitive reappraisal and behavioral regulation under stress, which in turn reduces anxiety (Audiffren and André, 2019). Thus, physical activity not only has a direct effect on mood but also enlarges an individual’s “psychological control resource pool,” supplying essential psychological capital for stress coping. Based on the theoretical and empirical evidence above, this study proposes the following hypothesis:

*H2*: Self-control plays a mediating role in the relationship between physical activity and anxiety among vocational college students.

### 1.3 The mediating role of mobile phone dependence between physical activity and anxiety among vocational college students

Mobile phone dependence significantly mediates the relationship between physical activity and anxiety. Mobile phone dependence denotes uncontrolled, excessive smartphone use that impairs psychosocial functioning and predicts higher anxiety levels (Dongmei et al., 2017; Yang et al., 2019). Its mechanisms are twofold. First, excessive phone use displaces opportunities to develop real-world social skills and to obtain social support, which can foster social alienation and anxiety (Kraut et al., 1998). Second, people may use their phones to escape real-world stress and negative emotions, creating a vicious cycle of “stress → escape → functional impairment → intensified anxiety” (Qin-xue et al., 2017).

Regular physical activity offers an effective intervention pathway for mobile phone dependence. At the behavioral level, structured exercise directly displaces the time and routines otherwise spent on passive phone-based entertainment. At the neuropsychological level, exercise stimulates release of endogenous substances such as endorphins, which produce natural sensations of pleasure and accomplishment (Korehpaz-Mashhadi et al., 2022) and thus furnish a healthy reward source independent of virtual stimuli. This diminished need for external rewards reduces motivation to seek immediate gratification via the phone. At the social-functioning level, especially in group-based contexts, physical activity generates high-quality real-world interactions that help rebuild social support networks and counteract the social alienation associated with mobile phone dependence.

Therefore, regular physical activity reduces mobile phone dependence by substituting healthy behaviors, providing intrinsic rewards, and fostering real-world social connections. This reduction disrupts the anxiety-maintaining mechanism of “virtual escape → real-world functional impairment” and thus alleviates anxiety symptoms. Based on this reasoning, we hypothesize:

*H3*: Mobile phone dependence mediates the relationship between physical activity and anxiety among vocational college students.

### 1.4 The chain mediating role of self-control and Mobile phone dependence between physical activity and anxiety

Physical activity may alleviate anxiety in vocational college students through a chain mediation pathway involving self-control and mobile phone dependence. This integrated model proposes that physical activity first strengthens psychological resources and then alters maladaptive behaviors. Specifically, regular physical activity, as a structured form of self-regulation training, can enhance an individual’s self-control capacity, thereby improving their ability to regulate impulses and pursue goals (Boat and Cooper, 2019). Enhanced self-control then helps individuals manage mobile phone use more effectively and inhibits excessive use driven by boredom, stress, or escapism, thus reducing mobile phone dependence (Zhongfeng, 2023). Reduced mobile phone dependence disrupts risk pathways such as “virtual immersion → weakening of real-world social functioning → increased social anxiety” and “continuous stimulation → sleep deprivation and cognitive overload → emotional dysregulation,” which in turn directly alleviates anxious emotions (Yang et al., 2019).

**Figure 1:**
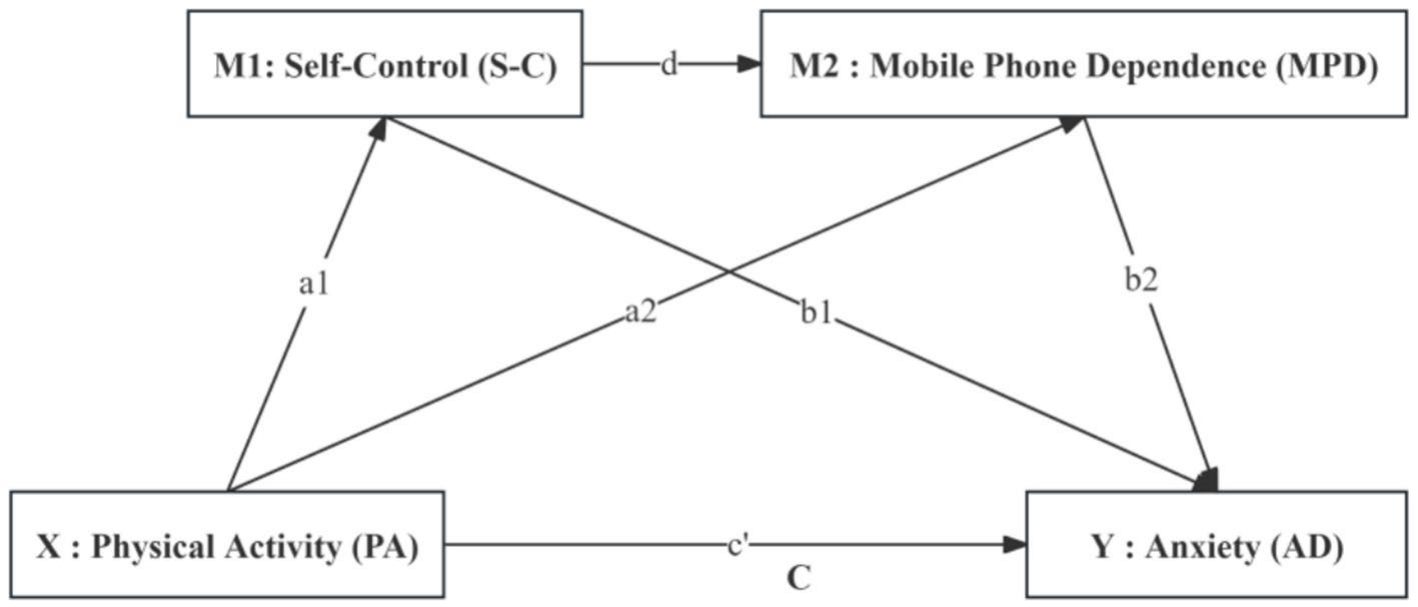
Hypothetical model diagram of the impact of physical exercise on anxiety among vocational college students.

Consequently, physical activity, by strengthening self-control, indirectly facilitates healthier management of mobile phone use. The resulting reduction in mobile phone dependence lessens the multiple sources of anxiety it generates and thereby reduces overall anxiety. This chain mediating effect underscores the multifaceted health benefits of physical activity in the digital age: it acts not only at the physiological level but also strengthens psychological resilience (self-control), which helps individuals resist maladaptive modern behaviors (mobile phone dependence) and thus addresses anxiety at its source (see Figure 1). On this basis, the present study proposes the following hypothesis:

*H4*: Self-control and mobile phone dependence play a chain mediating role in the relationship between physical activity and anxiety among vocational college students.

## 2 Research methods

### 2.1 Participants

This study was conducted between April and June 2025. The target population comprised students enrolled in vocational colleges in Shaanxi Province, China. A convenience cluster sampling method was used, selecting all students from 132 first- and second-year classes across four institutions. Data were collected via the “Questionnaire Star” platform, using a combination of online and offline procedures. After obtaining informed consent, students completed the questionnaires in approximately 15 min during breaks, under the supervision of the researchers and trained physical education instructors.

A total of 6,524 questionnaires were initially collected. Based on the statistical rule that the sample size should exceed five times the number of scale items (82 items in total), a minimum of 410 valid responses was required, and the initial sample satisfied this requirement. Invalid responses were then excluded according to these criteria: (1) completion time less than 300 s; (2) missing information or extreme outliers; (3) patterned responding or clearly contradictory answers. After exclusion, 5,991 valid questionnaires remained, yielding an effective response rate of 88.07%. The mean age of the final sample was 19.23 years (SD = 0.91). Detailed participant demographics are presented in Table 1.

**Table 1:** List of basic information of valid subjects (*N* = 5,991).

Variable	Category	*N* (%)	M ± SD	Variable	Category	*N* (%)	M ± SD
Gender	Male	3,443 (57.5%)	1.43 ± 0.49	Place of origin	Urban	1,103 (18.4%)	1.82 ± 0.39
	Female	2,548 (42.5%)			Rural	4,888 (81.6%)	
Ethnicity	Han ethnicity	5,896 (98.4%)	1.02 ± 0.12	Only child	No	4,774 (79.7%)	1.2 ± 0.40
	Other ethnicities	95 (1.6%)			Yes	1,217 (20.3%)	
Age	≤18	1,463 (24.4%)	19.23 ± 0.91	Body mass index	BMI<18.5	855 (14.3%)	23.26 ± 4.3
	19	2,167 (36.2%)			18.5 ≤ BMI<24.9	3,486 (58.2%)	
	20	1866 (31.1%)			25 ≤ BMI<29.9	969 (16.2%)	
	≥21	180 (3%)			BMI ≥ 30	681 (11.4%)	
Academic Year	Freshman	3,714 (62%)	1.38 ± 0.48				
	Sophomore	2,277 (38%)					

### 2.2 Measurement instruments

#### 2.2.1 Physical activity scale

This study utilized the Physical Activity Habit Scale for University Students, developed by Kun (2011). The scale consists of 26 items categorized into three dimensions: Exercise Behavior (Cronbach’s *α* = 0.892), Mindset (Cronbach’s α = 0.941), and Exercise Outcome (Cronbach’s α = 0.954). Each item is rated on a 5-point Likert scale, with higher scores reflecting greater consistency in exercise behavior and more favorable exercise outcomes. The *Kolmogorov–Smirnov (K–S)* test revealed a non-normal distribution (*D* = 0.123, *p* < 0.001) with a sample size of *N* = 5,991. The overall *Cronbach’s α* for the scale in this study was 0.969.

#### 2.2.2 Self-rating anxiety scale (SAS)

The SAS developed by Zung (1971) at Duke University Medical Center in 1971, assesses individuals’ subjective experiences of anxiety symptoms and serves as a critical reference for measuring anxiety severity. The scale comprises 20 self-assessment items.

#### 2.2.3 Self-control scale (SCS)

A revised Chinese version of the Self-Control Scale was utilized (Shuhua and Yongyu, 2008). This version comprises 19 items categorized into five dimensions: impulse inhibition, healthy habits, resisting temptation, focused work, and moderated recreation. The scale exhibits strong reliability and validity within Chinese populations and employs a 5-point Likert scale. The *Kolmogorov–Smirnov (K-S)* test revealed a non-normal distribution (*D* = 0.133). In this study, the overall *Cronbach’s α* for the scale was 0.892.

#### 2.2.4 Mobile phone addiction index (MPAI)

Chinese Version: The Chinese version of the MPAI, initially revised by Leung and Louis (2008) and validated for reliability and validity among Chinese university students by Hai et al. (2014), was employed in this study. This 17-item scale produces higher scores that indicate greater severity of mobile phone dependence. The *Kolmogorov–Smirnov (K-S)* test revealed a non-normal distribution (*D* = 0.131, *p* < 0.001). In this research, the overall *Cronbach’s α* was 0.955.

### 2.3 Data processing

(1) All valid data were imported into *SPSS* 27.0 for descriptive statistical analysis.
(2) *Spearman’s* correlation analysis, regression analysis, chain mediation analysis and moderating effect analysis are used, in which the chain mediation test adopts the procedure and methodology of the mediation effect test introduced by Zhonglin et al. (2004).
(3) Chained mediation analysis was conducted by using the Process (version 4.1) plug-in of Bootstrap method to test the mediation effect. When analyzing the indirect effects of variables using Process plug-in, the specific settings were as follows: set the model as No. 6 X = Physical Activity, M1 = Self-Control, M2 = Mobile Phone Dependence, Y = Anxiety situation; Bootstrap Samples = 5,000.

## 3 Findings

### 3.1 Common method Bias and homogeneity test

(1) Common Method Bias Test

Harman’s single-factor test (Dandan and Zhonglin, 2020) was conducted using an unrotated exploratory factor analysis on all variables. The results showed that 14 factors with eigenvalues greater than one were extracted, and the first factor accounted for 20.79% of the total variance, which is well below the critical threshold of 40%. This indicates that common method bias was not a serious concern in this study.

(2) Homogeneity Test

Given that cluster sampling was employed and students were nested within classes, there was a potential risk of non-independence in the data. To assess the clustering effect, null model analyses were first conducted on the four core variables. The results revealed that physical activity (ICC = 0.145) and mobile phone dependence (ICC = 0.216) exhibited clustering at the class level, whereas anxiety and self-control (ICC = 0.000) showed no clustering effect.

### 3.2 Overall situation of anxiety

The Self-Rating Anxiety Scale (SAS) was employed to assess the anxiety levels of 5,991 vocational college students. The results showed that the overall mean score of anxiety among the participants was 49.71 ± 11.01 (see Table 2). In accordance with the SAS scoring criteria outlined in the Handbook of Commonly Used Psychological Assessment Scales (Xiaoyang, 2011) and validated by Quanquan and Li (2012), raw scores were categorized as follows: < 50 indicates no anxiety, ≥ 50 indicates mild anxiety, ≥ 60 indicates moderate anxiety, and ≥ 70 indicates severe anxiety (Quanquan and Li, 2012). Based on these criteria, 2,751 participants (45.92%) were classified as having no anxiety, 1,515 (25.29%) as mild anxiety, 1,544 (25.77%) as moderate anxiety, and 181 (3.02%) as severe anxiety. The mean scores for each subgroup were as follows: 39.74 ± 6.47 for the no-anxiety group, 53.59 ± 3.07 for the mild anxiety group, 60.92 ± 1.98 for the moderate anxiety group, and 73.01 ± 3.94 for the severe anxiety group.

**Table 2:** Distribution of anxiety levels and associated demographic variables.

Anxiety level	*N*	Percentage	*M ± SD*
No anxiety	2,751	45.92%	39.74 ± 6.47
Mild anxiety	1,515	25.29%	53.59 ± 3.07
Moderate anxiety	1,544	25.77%	60.92 ± 1.98
Severe anxiety	181	3.02%	73.01 ± 3.94
Total	5,991	100%	49.71 ± 11.01

*N* = sample size; M ± SD = Mean ± Standard Deviation; SAS grouping criteria: No anxiety (< 50), mild anxiety (50–59), moderate anxiety (60–69), and severe anxiety (≥ 70).

Given the high prevalence of anxiety in the sample—with moderate and severe cases accounting for nearly 30% (28.79%) of the participants—it is of practical significance to explore its influencing factors and underlying mechanisms. To further clarify the relationships among physical activity, self-control, mobile phone dependence, and anxiety, a correlation analysis was conducted.

### 3.3 Correlation analysis

To investigate the relationships among the variables, *Spearman’s* rank correlation analysis was performed in this study (Table 3). The findings revealed that the core psychobehavioral variables exhibited strong internal associations. Anxiety demonstrated a strong positive correlation with mobile phone dependence (*r* = 0.581, 95% CI [0.564, 0.598], *p* < 0.001). Conversely, anxiety was strongly and negatively correlated with self-control (*r* = −0.510, 95% CI [−0.529, −0.490], *p* < 0.001). Additionally, mobile phone dependence showed a strong negative correlation with self-control (*r* = −0.512, 95% CI [−0.531, −0.492], *p* < 0.001). These results indicate that high levels of anxiety and mobile phone dependence are closely associated with low self-control.

**Table 3:** Correlation matrix between variables (*N* = 5,991).

Variable	1	2	3	4	5	6	7	8	9	10
1: Gender	1									
2: Ethnicity	0.002	1								
3: Age	−0.110^**^	0.035^**^	1							
4: BMI	−0.200^**^	−0.022	0.036^**^	1						
5: Grade	−0.037^**^	0.008	0.524^**^	−0.001	1					
6: Place of origin	0.050^**^	−0.043^**^	−0.031^*^	−0.017	−0.002	1				
7: Only child	−0.124^**^	−0.018	0.003	0.030^**^	0.011	−0.203^**^	1			
8: PA	−0.220^**^	0.027^*^	0.083^**^	−0.042^**^	0.092^**^	−0.008	0.016	1		
9: AD	0.023	0.000	−0.035^**^	0.018	−0.061^**^	−0.006	−0.003	−0.196^**^	1	
10: MPD	−0.011	−0.003	−0.036^**^	0.014	−0.072^**^	0.008	0.014	−0.104^**^	0.581^**^	1
11: S-C	0.016	0.004	0.037^**^	−0.055^**^	0.074^**^	0.011	−0.002	0.154^**^	−0.510^**^	−0.512^**^

Gender, ethnicity, age, grade, and BMI are categorical variables (e.g., male = 1, female = 2), and their correlation coefficients are rank correlation results. *p < 0.05, **p < 0.01 (two-tailed test).

### 3.4 Tests for mediating effects

To explore the relationships among physical activity, self-control, mobile phone dependence, and anxiety, we first conducted a hierarchical regression analysis based on correlation analysis, with the former three as independent variables and anxiety as the dependent variable, to examine their predictive effects on anxiety. Second, considering the potential non-independence arising from the overall sampling design, we performed a sensitivity analysis on the mediation model using cluster-robust standard errors (with class as the cluster variable).

Model 1 revealed that Physical Activity significantly negatively predicted Anxiety (*β* = −0.201, *t* = −15.918, *p* < 0.001), indicating that a one standard deviation increase in Physical Activity corresponded to a 0.201 standard deviation decrease in Anxiety. The model was significant (*F* = 253.405, *p* < 0.001) and accounted for 4.1% of the variance in Anxiety (*∆R*^2^ = 0.041).

In Model 2, after incorporating Self-Control, both predictors significantly negatively predicted Anxiety, collectively accounting for 27.2% of the variance (*∆R*^2^ = 0.272). Specifically, Physical Activity remained a significant negative predictor (*β* = −0.119, *t* = −10.600, *p* < 0.001), indicating that a one standard deviation increase in Physical Activity corresponded to a 0.119 standard deviation decrease in Anxiety. Self-Control also emerged as a significant negative predictor (*β* = −0.488, *t* = −43.635, *p* < 0.001), demonstrating a stronger effect, with a one standard deviation increase in Self-Control associated with a 0.488 standard deviation decrease in Anxiety. The overall model was significant, *F*(2, 5,988) = 1118.990, *p* < 0.001.

Model 3, which included Mobile Phone Dependence, significantly predicted Anxiety, accounting for 33.1% of the variance (*∆R*^2^ = 0.331). Physical Activity remained a significant negative predictor (*β* = −0.147, *t* = −13.829, *p* < 0.001), indicating that a one standard deviation increase in Physical Activity corresponded to a 0.147 standard deviation decrease in Anxiety. Mobile Phone Dependence was a significant positive predictor (*β* = 0.542, *t* = 51.014, *p* < 0.001), indicating that a one standard deviation increase in Mobile Phone Dependence was associated with a 0.542 standard deviation increase in Anxiety. The overall model was significant, *F*(2, 5,988) = 1482.927, *p* < 0.001.

In the final regression model (Model 4), all three predictors remained statistically significant, collectively accounting for 39.0% of the variance in anxiety scores (*△R*^2^ = 0.390, *F*(3, 5,987) = 1275.082, *p* < 0.001). After adjusting for the effects of the other covariates, physical activity maintained a significant negative association with anxiety (*β* = −0.113, *t* = −11.021, *p* < 0.001), indicating that a one standard deviation increase in physical activity corresponded to a 0.113 standard deviation decrease in anxiety. In contrast, mobile phone dependence exhibited a significant positive relationship (*β =* 0.400, *t* = 33.996, *p* < 0.001), with a one standard deviation increase in dependence associated with a 0.400 standard deviation increase in anxiety. Self-control also demonstrated a significant negative association (*β* = −0.285, *t* = −23.980, *p* < 0.001), such that a one standard deviation increase in self-control was linked to a 0.285 standard deviation decrease in anxiety. Among the three predictors, mobile phone dependence emerged as the strongest contributor to anxiety variance, followed by self-control.

All statistical tests reached significance (*p* < 0.05). All models satisfied regression assumptions: chi-square tests were acceptable, residuals showed independence (*D-W* ≈ 1.98) and approximate normality, and predictors exhibited no multicollinearity (VIF < 1.4) (see Table 4).

**Table 4:** Results of regression analysis of PA, S-C, and MPD on AD (*N* = 5,991).

Regression equation		Significance of regression coefficients						Overall model fit indices		
Predictor	Outcome	*B*	*SE^a^*	*β*	*t*	*p*	*D-W*	*R*	*△R* ^2^	*F*
PA	AD(1)	−0.154	0.011	−0.201	−15.919	<0.001	1.923	0.201	0.041	*F*(1,5,989) = 253.405**
PA	AD(3)	−0.091	0.010	−0.119	−10.600	<0.001	1.980	0.522	0.272	*F*(2,5,988) = 1118.990**
S-C		−0.452	0.012	−0.488	−43.635	<0.001				
PA	AD(2)	−0.112	0.011	−0.147	−13.829	<0.001	1.977	0.576	0.331	F(2,5,988) = 1482.927**
MPD		0.400	0.008	0.542	51.014	<0.001				
PA	AD(4)	−0.086	0.009	−0.113	−9.42	<0.001	1.994	0.624	0.390	*F*(3,5,987) = 1132.18***
S-C		−0.264	0.013	−0.285	−20.18	<0.001				
MPD		0.295	0.010	0.400	29.26	<0.001				
PA	MPD	−0.104	0.016	−0.101	−7.836	<0.001	1.953	0.101	0.010	*F*(1,5,989) = 61.397**
	S-C	0.140	0.014	0.170	13.333	<0.001	1.917	0.170	0.029	*F*(1,5,989) = 177.777**
MPD	AD	0.411	0.011	0.557	51.857	<0.001	1.973	0.557	0.310	*F*(1,5,989) = 2689.184**
	S-C	−0.640	0.019	−0.512	−46.129	<0.001	1.954	0.512	0.262	*F*(1,5,989) = 2127.906**
S-C	AD	−0.471	0.014	−0.508	−45.682	<0.001	1.983	0.508	0.258	*F*(1,5,989) = 2086.823**

*p < 0.05, **p < 0.01, same below; (1) Model 1, (2) Model 2, (3) Model 3, (4) Model 4, B = unstandardized coefficient, β = standardized coefficient; SE^a^ = heteroscedasticity-consistent standard error (HC3).

Based on the mediation effect testing process proposed by Zhonglin et al. (2004):

In the first step, the effect of physical activity on anxiety was tested (*C* = −0.201, t = −15.919, *p* < 0.001), establishing a direct association between physical activity and reduced anxiety.

**Figure 2:**
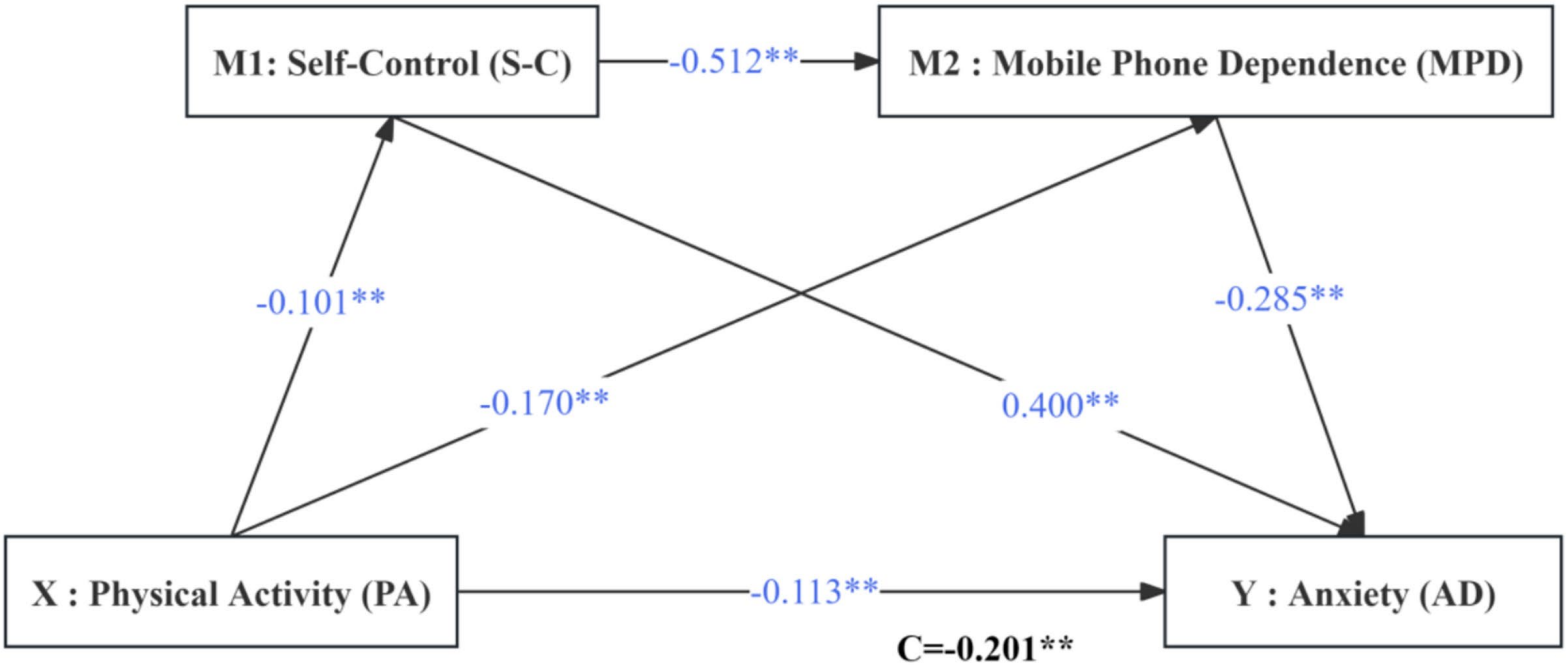
The influence of physical activity on anxiety.

In the second step, the estimated coefficients were a_1_ = −0.101 and a_2_ = 0.170, with b_1_ = 0.400 and b_2_ = −0.285; all four coefficients were statistically significant, and the indirect effect was significant, yielding the model and path relationships shown in Figure 2.

In the third step, the PROCESS macro (Model 6) was employed to examine the serial mediating roles of self-control and mobile phone dependence in the relationship between physical activity and anxiety. Heteroscedasticity-consistent standard errors (HC3) and 5,000 bootstrap samples were used to estimate indirect effects and their confidence intervals. The results are presented in Table 5.

**Table 5:** Direct and indirect effects, Bootstrap test, and relative mediation effects.

Impact pathways	*B*	Boot *SE*	95%CI(*B*)	*β*_ind_	Boot *SE*	95% CI (*β*_ind_)
Total effect	−0.154	0.011^a^	[−0.176, −0.132]	−0.202	0.015^a^	[−0.231, −0.173]
Direct effect	−0.086	0.009^a^	[−0.104, −0.068]	−0.113	0.012^a^	[−0.136, −0.089]
Total indirect effect	−0.068	0.008	[−0.083, −0.053]	−0.089	0.010	[−0.108, −0.069]
Ind1: X → M1 → Y	−0.037	0.004	[−0.046, −0.029]	−0.048	0.005	[−0.059, −0.038]
Ind2: X → M2 → Y	−0.040	0.007	[−0.053, −0.028]	−0.054	0.009	[−0.071, −0.037]
Ind3: X → M1 → M2 → Y	−0.027	0.003	[−0.032, −0.021]	−0.035	0.004	[−0.042, −0.027]
C1: Ind1 vs. Ind2	−0.033	0.007	[−0.045, −0.019]	−0.043	0.008	[−0.060, −0.027]
C2: Ind1 vs. Ind3	−0.011	0.003	[−0.016, −0.005]	−0.014	0.004	[−0.021, −0.007]
C3: Ind2 vs. Ind3	0.022	0.006	[0.010, 0.034]	0.030	0.008	[0.015, 0.045]

B, unstandardized coefficient; β_ind_, completely standardized coefficient. Boot SE and 95% CI are based on 5,000 bootstrap samples. X, Physical Activity (PA); M1, Self-Control (S-C); M2, Mobile Phone Dependence (MPD); Y, Anxiety (AD). ^a^Standard errors for total and direct effects are heteroscedasticity-consistent (HC3). Ind1 = PA–SC–AD; Ind2 = PA–MPD–AD; Ind3 = PA–SC–MPD–AD. C1, C2, and C3 represent pairwise contrasts of the corresponding indirect effects. Relative mediation effects are calculated based on unstandardized indirect effects.

#### 3.4.1 Total and direct effects

Physical activity exhibited a significant negative total effect on anxiety (*B* = −0.154, 95% CI [−0.176, −0.132]; *β*_ind_ = −0.202, 95% Cl [−0.231, −0.173). After including the two mediators, the direct effect of physical activity on anxiety remained significant (*B* = −0.086, 95% CI [−0.104, −0.068]; *β*_ind_ = −0.113, 95% CI [−0.136, −0.089]), indicating that self-control and mobile phone dependence partially mediated this relationship.

The total indirect effect was *B* = −0.068 (95% CI [−0.083, −0.053]; *β*_ind_ = −0.089, 95% CI [−0.108, −0.069]). Further analysis revealed three significant indirect pathways. In terms of standardized effect sizes (see Table 5), Path 2 exhibited the largest effect (*β*_ind_ = −0.054, 95% CI [−0.071, −0.037]), followed by Path 1 (*β*_ind_ = −0.048, 95% CI [−0.059, −0.038]) and Path 3 (*β*_ind_ = −0.035, 95% CI [−0.042, −0.027]). This suggests that in the association between physical activity and anxiety, the mediating role of mobile phone dependence alone contributed the most, followed by self -control alone, while the serial mediation path contributed a smaller yet significant portion. Specifically:

##### 3.4.1.1 Path 1: Physical activity → self-control → anxiety

Physical activity significantly and positively predicted self-control (*B* = 0.140, *p* < 0.001; *β* = 0.170), and self-control significantly and negatively predicted anxiety (*B* = -0.264, *p* < 0.001; *β* = −0.284). The indirect effect was *B* = −0.037, 95% CI [−0.046, −0.029], (*β*_ind_ = −0.048, 95% CI [−0.059, −0.038]), with the confidence interval excluding zero, indicating a significant mediating effect of self-control alone.

##### 3.4.1.2 Path 2: Physical activity → mobile phone dependence → anxiety

Physical activity significantly and negatively predicted mobile phone dependence (*B* = −0.105, *p* < 0.001; *β* = −0.101), and mobile phone dependence significantly and positively predicted anxiety (*B* = 0.295, *p* < 0.001; *β* = 0.400). The indirect effect was *B* = −0.040, 95% CI [−0.053, −0.028], (*β*_ind_ = −0.054, 95% CI [−0.071, −0.037]), with the confidence interval excluding zero, indicating a significant mediating effect of mobile phone dependence alone.

##### 3.4.1.3 Path 3: Physical activity → self-control → mobile phone dependence → anxiety

The indirect effect was *B* = −0.027, 95% CI [−0.032, −0.021], (*β*_ind_ = −0.035, 95% CI [−0.042, −0.027]), with the confidence interval excluding zero, confirming a significant serial mediation effect through self-control and mobile phone dependence.

#### 3.4.2 Contrasts among indirect effects

Pairwise comparisons revealed that the mediating effect of self-control alone (Path 1) was significantly stronger than that of mobile phone dependence alone (Path2) (*C1* = −0.033, 95% CI [−0.045, −0.019]; *β*_ind_ = −0.043, 95% CI [−0.060, −0.027]), and significantly stronger than the serial mediation effect (Path 3) (*C2* = −0.011, 95% CI [−0.016, −0.005; *β*_ind_ = −0.014, 95% CI [−0.021, −0.007]). Furthermore, the mediating effect of mobile phone dependence alone (Path 2) was significantly stronger than the serial mediation effect (Path 3) (*C3* = 0.022, 95% CI [0.010, 0.034; *β*_ind_ = 0.030, 95% CI [0.015, 0.045]).

In summary, physical activity is associated with lower anxiety both directly and indirectly through its relations with self-control and mobile phone dependence. Specifically, the association between physical activity and anxiety is partially explained by the independent and sequential effects of self-control and mobile phone dependence, with varying magnitudes across these pathways.

### 3.5 Robustness check

To rule out potential confounding effects of demographic variables, gender, age, BMI, grade, origin, and only-child status were included as covariates in the serial mediation model, allowing them to influence both the mediators and the outcome variable. The model was re-estimated using the same analytical approach. The results showed that after controlling for these demographic variables, the direction and significance of all core path coefficients, as well as the confidence intervals of the indirect effects, remained consistent with the main findings, indicating that the conclusions of this study are robust. Detailed results are presented in Supplementary Table S1 of the Supplementary Material.

## 4 Discussion

This study identifies a mediating pathway in which mobile phone dependence and self-control elucidate the relationship between physical activity and anxiety among vocational college students. By examining the mechanisms through which physical activity mitigates anxiety, this research enhances the theoretical understanding of anxiety influences within student populations and advances the exploration of how physical activity fosters psychological resilience. Practically, it offers strategies for promoting physical activity, decreasing mobile phone dependence, improving self-control, and alleviating anxiety among vocational college students.

### 4.1 Effects of physical activity (PA) on anxiety (AD)

The results of this study reveal a significant negative correlation between physical activity and anxiety levels among vocational college students (*r* = −0.196, *p* < 0.01). Moreover, the negative predictive effect of physical activity on anxiety remained significant after accounting for the mediating variables. This finding is consistent with conclusions drawn from large-scale surveys and meta-analyses, which consistently report an inverse association between physical activity and anxiety symptoms (Stubbs et al., 2017; Wipfli et al., 2008), thereby supporting Hypothesis *H1* of this study.

The relationship between physical activity and anxiety is complex. First, physical activity can directly and effectively lower anxiety levels, with effect sizes reaching a moderate magnitude (Lin and Gao, 2023). Various forms of exercise have demonstrated interventive efficacy (Gordon et al., 2021), with intensity, frequency, and duration being critical variables that influence this direct alleviation effect (Wipfli et al., 2008). Second, physical activity enhances key psychological resources, such as self-control capacity, psychological resilience, mindfulness, and emotion regulation, thereby establishing an internal buffer system against anxiety (Manluo et al., 2025; Zhao et al., 2025). For vocational college students, whose anxiety often revolves around “skill competence” and “career future,” the development of these psychological qualities directly addresses challenges related to practical training pressure, workplace competition, and identity transition.

Moreover, physical activity functions as a “non-evaluative” social and experiential activity, providing students with essential psychological detachment. Engaging in group-based physical activity offers opportunities for positive psychological support from peers, alleviates concerns about negative evaluation and social fears, and mitigates negative psychological states such as experiential avoidance. This engagement helps counteract emotional exhaustion resulting from academic comparison or social bias (Peng et al., 2025). These findings support the perspective of positive psychological development, indicating that physical activity can positively influence students’ overall emotional growth and personality maturation by fostering positive affective experiences and social connections.

### 4.2 The mediating role of self-control

The results of this study offer empirical evidence for the mediation pathway of “physical activity → self-control → anxiety” (*β* = −0.119, *p* < 0.001; *β* = −0.488, *p* < 0.001; Ind1: *B* = −0.037, *β*_ind_ = −0.048), aligning with prior research (Diamond and Ling, 2016). From a neuro-mechanistic perspective, regular physical activity effectively promotes both the structural and functional optimization of the prefrontal cortex, particularly enhancing core executive functions such as inhibitory control and cognitive flexibility (Zhiming et al., 2018). This optimization establishes a neuroplastic foundation for improving self-control capacity. The regression analysis results of this study (*β* = 0.170, *p* < 0.001) confirm that physical activity can significantly and positively predict self-control levels. This conclusion aligns with the experimental findings of Muraven (2010), who reported that aerobic and endurance training significantly improved individuals’ self-control performance (*p* < 0.01), thereby further supporting the role of physical activity in building self-control resources.

This study importantly verifies the mediating role of self-control in the relationship between physical activity and anxiety among vocational college students (*H2* supported). According to resource depletion theory, experiences of anxiety in both life and learning contexts often lead to accelerated depletion of self-control resources. When these resources fall below a critical threshold, individuals may enter a state of ego depletion, which intensifies anxiety (Kashdan et al., 2011). Physical activity serves as an effective means of restoring and reinforcing these resources. By enhancing an individual’s self-control capacity, it reduces the rate of resource depletion and strengthens emotional regulation and interpersonal adaptation abilities (Xinyu, 2020). Individuals with high self-control not only manage emotional responses more effectively in social situations but also exhibit stronger interpersonal skills and greater subjective well-being (Shuhua and Yongyu, 2008). Collectively, these factors enhance motivation for social participation and positive experiences, thereby reducing anxiety levels (Tunç and Akandere, 2020). Consequently, physical activity not only directly fosters the development of self-control but also indirectly alleviates anxiety among vocational college students through this psychological resource as a mediating pathway.

In conclusion, this study empirically elucidates the mediating role of self-control in the relationship between physical activity and anxiety. It underscores the substantial practical importance of systematically enhancing adolescents’ self-control capacities through physical activity as a strategy for the prevention and intervention of anxiety.

### 4.3 The mediating role of Mobile phone dependence

This study confirms that mobile phone dependence mediates the relationship between physical activity and anxiety among vocational college students. The findings indicate that physical activity alleviates anxiety both directly and indirectly by reducing mobile phone dependence (Ind2: *B* = −0.040, *β*_ind_ = −0.054), thereby supporting Hypothesis *H3*.

Physical activity enhances the function of the prefrontal cortex, thereby improving an individual’s cognitive control and capacity for impulse inhibition. This enhancement provides a neural basis for mitigating the tendency toward excessive phone use (Xie et al., 2025). Simultaneously, as a proactive health behavior, physical activity occupies leisure time, presenting a constructive alternative to phone use (Pirwani et al., 2025). This finding aligns with the significant negative correlation observed in this study (*r* = −0.104, *p* < 0.01) and is further supported by meta-analyses indicating that exercise interventions may offer unique benefits in reducing mobile phone dependence (Qiong et al., 2023).

The findings of this study indicate that mobile phone dependence significantly and positively predicts anxiety (*r* = 0.581, *β* = 0.542, *p* < 0.001). This relationship often creates a vicious cycle: mobile phone dependence can directly intensify anxiety by fostering social comparison and undermining real-world support systems (Kim et al., 2021); conversely, individuals experiencing anxiety may utilize their phones as a means of escape, thereby reinforcing dependent behaviors (Elkin et al., 2025). Physical activity disrupts this cycle by diminishing dependence behaviors, thus providing an effective strategy for alleviating anxiety.

In summary, this study uncovers the mediation pathway of “physical activity → reduced mobile phone dependence → alleviated anxiety” offering empirical support for utilizing exercise interventions to tackle phone dependence and anxiety in students. Further investigation is needed to explore the underlying psychological mechanisms, including whether the impact is mediated by enhancing general emotional well-being (e.g., loneliness, depression) (De Berardis et al., 2009).

### 4.4 The chain mediating role of self-control and Mobile phone dependence

Physical activity can directly enhance self-control capacity, a benefit attributed to its role in promoting neural efficiency related to executive functions (Xie et al., 2025). This strengthened self-control equips individuals to resist immediate temptations more effectively and manage their usage behaviors, thereby serving as a crucial psychological resource for reducing mobile phone dependence (Wang et al., 2024; Ye et al., 2025). Ultimately, the reduction in mobile phone dependence weakens its anxiety-elevating effects, which operate through mechanisms such as sleep disruption, social comparison, and increased stress (Kim et al., 2021). Consequently, these four factors form a sequential pathway: “physical activity → enhanced self-control → reduced mobile phone dependence → alleviated anxiety.” The results of this study validate this pathway: self-control and mobile phone dependence exhibited significant individual mediating effects between physical activity and anxiety (*β* = 0.488, *p* < 0.001, *β*_ind_ = −0.048, 95% CI [−0.059, −0.038]; *β* = 0.542, *p* < 0.001, *β*_ind_ = −0.054, 95% CI [−0.071, −0.037]). Furthermore, they confirm the effectiveness of the chain mediation pathway proposed in Hypothesis H4: “physical activity → increased self-control → reduced mobile phone dependence → alleviated anxiety” (*B* = −0.027, 95% CI [−0.032, −0.021], *β*_ind_ = −0.035, 95% CI [−0.042, −0.027]). This pathway clearly delineates the progressive psychological process through which positive health behaviors influence emotional states.

This study theoretically employs a chain mediation model to integrate the pathways of “resource acquisition (self-control)” and “problem behavior reduction (mobile phone dependence),” systematically elucidating how physical activity contributes to long-term emotional health benefits. This approach surpasses the explanatory capacity of a single-mediator model, offering a more nuanced framework for understanding the intricate transmission mechanisms between health behaviors and mental health. Additionally, it resonates with and expands the application of Conservation of Resources Theory and the Strength Model of Self-Control in the digital age.

Empirical results demonstrate that the predictive effect size of mobile phone dependence on anxiety (*β* = 0.557) is significantly greater than that of other variables. This effect size persists at a relatively high level (*β* = 0.400) in the comprehensive model that includes all variables, indicating that mobile phone dependence is a major problematic behavior contributing to anxiety. From a practical application standpoint, this suggests that interventions aimed at reducing mobile phone dependence may provide the most substantial benefits in alleviating anxiety.

The observation that physical activity can significantly and positively predict levels of self-control (Du et al., 2025) is consistent with the findings of this study, which demonstrated a positive correlation between physical activity and self-control (*r* = 0.154, *p* < 0.001). Furthermore, the strong negative correlation between mobile phone dependence and self-control (*r* = −0.512, *p* < 0.01), the positive correlation between mobile phone dependence and anxiety (*r* = 0.581, *p* < 0.01), and the significant negative correlation between self-control and anxiety (*r* = −0.510, *p* < 0.01) offer important insights into the intricate relationships among these three variables. Specifically, physical activity may indirectly mitigate mobile phone dependence behaviors by enhancing self-control, which in turn may reduce the risk of anxiety.

Regular physical activity serves as a systematic approach for vocational college students to enhance emotional health by improving self-control and reducing dependence on mobile phones. Based on this premise, a tiered intervention framework can be developed for practical implementation. This framework prioritizes the cultivation of self-control as the primary objective, employs regular physical activity as the foundational method, and focuses on the prevention and management of mobile phone dependence as a critical area of concern.

## 5 Conclusion

This study utilized structural equation modeling and the Bootstrap method to systematically investigate the internal relationships and underlying mechanisms among physical activity, self-control, mobile phone dependence, and anxiety in vocational college students. The primary conclusions are as follows:

(1) Correlational Analysis among Variables

Significant pairwise correlations were identified among physical activity, self-control, mobile phone dependence, and anxiety in vocational college students. Specifically, physical activity exhibited a significant positive correlation with self-control and significant negative correlations with both mobile phone dependence and anxiety. Mobile phone dependence displayed a significant positive correlation with anxiety and a significant negative correlation with self-control. Additionally, self-control revealed a significant negative correlation with anxiety.

(2) Individual Mediating Effects

Mediation analyses indicated that both self-control and mobile phone dependence served as significant individual mediators in the relationship between physical activity and anxiety among vocational college students. The effect size for self-control was −0.040, while that for mobile phone dependence was −0.034. These findings further elucidate the dual-pathway mechanism by which physical activity affects anxiety.

(3) Chain Mediating Effect

Self-control and mobile phone dependence acted as sequential mediators between physical activity and anxiety, establishing the pathway: “physical activity → self-control → mobile phone dependence → anxiety.” The total indirect effect of this chain was −0.014. These findings offer a novel theoretical perspective for elucidating the intricate mechanisms through which physical activity influences anxiety and provide a practical foundation for interventions aimed at reducing anxiety among vocational college students through physical activity.

The research confirmed the explanatory power of a mediation model that incorporates self-control and mobile phone dependence in the relationship between physical activity and anxiety among vocational college students. Specifically, mobile phone dependence impeded the anxiety-reducing benefits of physical activity. Conversely, self-control, regarded as a form of psychological capital gained through resource acquisition, not only served as a significant independent mediator in this relationship but also effectively countered the adverse mediating influence of mobile phone dependence.

### 5.1 Implications for practice

This study elucidates the pathway of “physical activity → self-control → mobile phone dependence → anxiety”. This finding indicates that future mental health promotion initiatives should extend beyond single-level interventions to embrace an integrated “behavioral-psychological-environmental” systemic strategy. Specifically, it is essential to acknowledge the comprehensive value of physical activity in shaping psychological and behavioral management. Methodologically, synergistic programs that combine physical activity, cognitive training, and digital literacy education can be developed. For instance, physical education classes might incorporate team challenges that require persistence and focus, thereby embedding self-control cultivation into specific tasks. Students could also be encouraged to establish micro-habits, such as a daily 10-min rope-skipping routine, and to utilize stretching as an alternative to immediately reaching for their phones. In terms of the environment, fostering a campus ecology that supports active lifestyles and real-world social interaction is crucial. This could be accomplished by adding convenient exercise points in training and dormitory areas, establishing class agreements for “phone-free breaks,” and considering physical activity participation in merit-based evaluations. The essence of this multi-level systemic intervention lies in translating the cultivation of abstract psychological resources into observable, participatory actions. This approach can effectively disrupt the cycle of “low self-control – high dependence – high anxiety” and enhance students’ overall health.

### 5.2 Limitations and future directions

First, the sample was restricted to vocational college students from Shaanxi Province, which limits the generalizability of the findings. This population may differ from regular high school or undergraduate students in learning patterns and stressors, and whether the results hold for other regions, educational stages, or cultural contexts requires further validation. Future research should expand the geographical coverage and include more diverse institutional types for comparison. Second, all variables were assessed using self-report measures, which are susceptible to social desirability bias, recall bias, and common method bias. Although statistical tests indicated that common method bias was not a serious concern, the findings lacked objective indicators (e.g., accelerometer data, usage logs, clinical interviews) to corroborate the measurements. Future studies should incorporate multi-source data to enhance the objectivity of assessments. Third, while the theoretical framework integrated self-control theory and research on mobile phone dependence, vocational students face unique stressors such as employment pressure and social prejudice that were not adequately controlled in this study. Moreover, the mediation effect sizes were relatively small (*β* = 0.04–0.08), suggesting the presence of other explanatory pathways (e.g., social support, emotion regulation). Future research should include more contextually relevant variables (e.g., employment anxiety, internship stress) to more accurately elucidate the underlying mechanisms. Fourth, the cross-sectional design precludes causal inferences. Reverse or bidirectional relationships may exist among variables (e.g., anxiety may reduce physical activity engagement). Future research should employ longitudinal designs or experimental interventions to verify temporal relationships and causal directions.

## Data Availability

The raw data supporting the conclusions of this article will be made available by the authors, without undue reservation.
