# Supplementary material for: Effects of physical activity on anxiety phenomenon in vocational college students–chain mediation of self-control and mobile phone dependence
Source: Front Psychol. 2026 Apr 17;17:1786180. doi: 10.3389/fpsyg.2026.1786180 (PMC13132731; doi:10.3389/fpsyg.2026.1786180)
Supplement: Supplementary file 1 [file Data_Sheet_1.pdf]

Table 1

Path Coefficients of the Serial Mediation Model After Controlling for Demographic Variables

**Predictor**	**Outcome Variable**	*B*	*SE*[a]	*β*	*t*	*p*	**△*R*²**	***F***
PA	S-C	0.139	0.014	0.169	9.86	<0.001	0.036	$F(7,5983)=20.76^{***}$
PA	MPD	-0.094	0.016	-0.091	-7.71	<0.001	0.008	$F(8,5982)=191.36^{***}$
S-C		-0.638	0.019	-0.508	-33.38	<0.001		
PA	AD	-0.086	0.009	-0.113	-9.12	<0.001	0.390	$F(9,5981)=381.28^{***}$
S-C		-0.264	0.013	-0.284	-20.11	<0.001		
MPD		0.295	0.010	0.400	29.21	<0.001		

Note: ***p < 0.001 ; *B* = unstandardized coefficient, *β* = standardized coefficient; *SE*[a] = heteroscedasticity-consistent standard error (HC3).

Table 2

Direct and Indirect Effects After Controlling for Demographic Variables

**Impact pathways**	*B*	**Boot SE**	**95%CI**	**Relative Mediation Effect**
Total Effect	-0.153	0.012[a]	[-0.176, -0.130]	-
Direct Effect	-0.086	0.009[a]	[-0.105, -0.068]	-
Total Indirect Effect	-0.066	0.008	[-0.083, -0.051]	100%
Ind1:X→M1→Y	-0.037	0.004	[-0.045, -0.029]	38.2%
Ind2:X→M2→Y	-0.031	0.003	[-0.039, -0.016]	40.9%
Ind3:X→M1→M2→Y	-0.026	0.003	[-0.032, -0.021]	20.9%
C1:Ind1 vs Ind2	-0.033	0.007	[-0.046, -0.021]	-
C2:Ind1 vs Ind3	-0.011	0.003	[-0.016, -0.005]	-
C3:Ind2 vs Ind3	0.023	0.006	[0.012, 0.035]	-

Note: B = unstandardized coefficient; Boot SE = bootstrap standard error; CI = confidence interval. PA: Physical Activity; S-C: Self-Control; MPD: Mobile Phone Dependence; AD: Anxiety.Ind1= PA - SC - AD; Ind2= PA -MPD - AB; Ind3= PE - SC - MPD - AB. C1 = Ind1 vs Ind2; C2 = Ind1 vs Ind3; C3 = Ind2 vs Ind3.

Table 3

Comparison of Direct and Indirect Effects Before and After Adding Covariates

**Indicator**	**Without Covariates**	**With Covariates**	**Change**
PA → S-C	0.140***	0.139***	Coefficient largely unchanged, still significant
PA → MPD	-0.104***	-0.094***	Remained non-significant
S-C → MPD	-0.640***	-0.638***	Coefficient largely unchanged, still significant
S-C → AD	-0.264***	-0.264***	Coefficient unchanged, still significant
MPD → AD	0.295***	0.295***	Coefficient unchanged, still significant
Total Effect	-0.154***	-0.153***	Coefficient largely unchanged, still significant
Direct Effect	-0.086***	-0.086***	Coefficient unchanged, still significant
Total Indirect Effect	-0.068 [-0.083, -0.053]	-0.066 [-0.083, -0.051]	Slightly decreased, still significant
Ind1	-0.037 [-0.046, -0.029]	-0.037 [-0.045, -0.029]	Identical
Ind2	-0.040 [-0.053, -0.028]	-0.031 [-0.039, -0.016]	Largely unchanged, still significant
Ind3	-0.027 [-0.032, -0.021]	-0.026 [-0.032, -0.021]	Largely unchanged, still significant
C1	-0.033 [-0.045, -0.019]	-0.033 [-0.046, -0.021]	Identical
C2	-0.011 [-0.016, -0.005]	-0.011 [-0.016, -0.005]	Identical
C3	0.022 [0.010, 0.034]	0.023 [0.012, 0.035]	Identical
Anxiety Equation R^2	0.390	0.390	Identical

Note. PA = Physical Activity, S-C = Self-Control, MPD = Mobile Phone Dependence, AD = Anxiety. Values in brackets represent 95% confidence intervals. ***p < 0.001. The "With Covariates" model controlled for gender, age, BMI, grade, origin, and only-child status. Ind1 = PA → S-C → AD; Ind2 = PA → MPD → AD; Ind3 = PA → S-C → MPD → AD; C1 = Ind1 vs Ind2; C2 = Ind1 vs Ind3; C3 = Ind2 vs Ind3.
